# Editorial: Microglia and tissue macrophages in pain

**DOI:** 10.3389/fimmu.2025.1666972

**Published:** 2025-08-07

**Authors:** Darian Mohsenin, Jessica Yu, Zhonghui Guan, Xiaobing Yu

**Affiliations:** ^1^ Department of Anesthesia and Perioperative Care, University of California, San Francisco, San Francisco, CA, United States; ^2^ Center for Pain Medicine, University of California, San Francisco, San Francisco, CA, United States

**Keywords:** microglia, macrophage, neuropathic pain, DRG, spinal cord, neuroinflammation

Following injury or diseases of the somatosensory nervous system, neuropathic pain is usually long-lasting and manifested with either negative symptoms such as loss of sensation or positive symptoms characterized by allodynia or hyperalgesia in a neuroanatomically plausible distribution (1). Neuropathic pain can be further divided into peripheral and central pathology. Common peripheral neuropathic pain includes trigeminal neuralgia, peripheral nerve injury-induced chronic neuropathic pain, postherpetic neuralgia, etc., whereas central neuropathic pain can coexist with lesions of the central nervous system such as traumatic brain and spinal cord injury, stroke and multiple sclerosis (MS) (2). With prevalence varying from 6.9% to 10% (3), neuropathic pain profoundly impacts human well-being, both physically and psychologically, with substantial health care costs. To date, managing neuropathic pain remains an unmet clinical challenge.

Over the past decades, there has been an exponential growth of both preclinical and clinical research on neuropathic pain. Insights of pain processing mechanisms have come from studying peripheral nerve injury induced neuropathic pain models (4), and neuroimmune interactions are increasingly recognized as an essential driver of neuropathic pain (5). It is well known that spinal microglia contribute to the induction and maintenance of neuropathic pain following peripheral nerve injury (6–9). Moreover, microglia also play a critical role in inducing sensitization of the dorsal horn pain processing circuitry by releasing pro-inflammatory mediators, such as interleukin-1β (IL-1β), tumor necrosis factor-alpha (TNF-ɑ), and prostaglandin E2 (PGE_2_) (6). Notably, the contribution of microglia signaling to nerve-injury induced neuropathic pain is sexually dimorphic (10). Targeting spinal microglial cells to reduce injury-induced mechanical hypersensitivity is only effective in male mice and has little impact in female mice. On the other hand, a reciprocal interaction between sensory neurons and macrophages contributes to both acute (11) and persistent injury-induced neuropathic and inflammatory pain, in both sexes (12–14). A recent nociceptor-immune interactome study further predicts that macrophages are the strongest immune cell interactors of sensory neurons in the context of tissue injury (15).

Through preclinical studies, many potential analgesic targets implicated in neuropathic pain in rodents have been identified*.* Unfortunately, most molecular candidates have failed to translate to the clinic. It has been increasingly debated among pain researchers and clinicians whether preclinical animal models reliably recapitulate human therapeutic responses, given interspecies biological differences (16, 17) and unreliable outcome measures (18).

In this Research Topic of *Frontiers in Immunology*, Dhir et al. set out to identify knowledge gaps in the research of investigating spinal microglia changes in murine models of neuropathic pain. Their comprehensive review found that changes in microglia and pain behavior were only acutely recorded up to 2 weeks after nerve injury in most of the 258 peer-reviewed articles. The findings highlight the need to study chronic pain models. Moreover, the authors call for the inclusion of both sexes in future chronic pain studies.

Aiming for developing novel nonopioid pain treatment, Ruan et al. focused on ozone therapy in chronic constriction injury of the sciatic nerve animals. The authors demonstrated that Ozone significantly alleviated neuropathic pain by promoting macrophage efferocytosis and suppressing neuroinflammation through the AMPK/Gas6-MerTK/SOCS3 signaling pathway. Although Ozone therapy has been proposed as an adjunct pain treatment for its antimicrobial, anti-inflammatory properties (19), its efficacy and long-term safety remains to be determined.

With etiology poorly understood, neuropathic pain commonly occurs in 20% to 50% of MS patients (20, 21). Ouyang et al. now explored the risk factors of neuropathic pain in MS. The authors concluded that the neuropathic pain risk parallels with the chronicity of spinal lesions in MS patients. Meanwhile, extended cervical and thoracic lesions were independent risk factors of neuropathic pain. The researchers further encouraged early attention to spinal cord lesions to improve pain management in patients with MS.

Chemotherapy-induced neuropathic pain (CINP) is also common among cancer patients. Moraes et al. investigated the role of spinal high-mobility group box 1 (HMGB1) in CINP. Their findings suggest that targeting the release of HMGB1 during the early stages of CINP may relieve neuropathic pain by reducing pro-inflammatory cytokines release from spinal microglia.

Taken together, this Research Topic aims to addressing the gaps in chronic pain research, emphasizing longitudinal studies and inclusive experimental designs. The findings further underscore the complementary roles of microglia and macrophages in the development and resolution of neuropathic pain. Integrating insights from both compartments will hold promise for translating neuroimmune findings into the clinic and improving pain management.

## References

[1] Finnerup NB Haroutounian S Kamerman P Baron R Bennett DLH Bouhassira D. Neuropathic pain: an updated grading system for research and clinical practice. Pain. Aug. (2016) 157:1599–606. doi: 10.1097/j.pain.0000000000000492, PMID: 27115670 PMC4949003

[2] Scholz J Finnerup NB Attal N Aziz Q Baron R Bennett MI. The IASP classification of chronic pain for ICD-11: chronic neuropathic pain. Pain. Jan. (2019) 160:53–9. doi: 10.1097/j.pain.0000000000001365, PMID: 30586071 PMC6310153

[3] van Hecke O Austin SK Khan RA Smith BH Torrance N. Neuropathic pain in the general population: a systematic review of epidemiological studies. Pain. Apr. (2014) 155:654–62. doi: 10.1016/j.pain.2013.11.013, PMID: 24291734

[4] Fiore NT Debs SR Hayes JP Duffy SS Moalem-Taylor G. Pain-resolving immune mechanisms in neuropathic pain. Nat Rev Neurol Apr. (2023) 19:199–220. doi: 10.1038/s41582-023-00777-3, PMID: 36859719

[5] Basbaum AI Bautista DM Scherrer G Julius D. Cellular and molecular mechanisms of pain. Cell Oct 16. (2009) 139:267–84. doi: 10.1016/j.cell.2009.09.028, PMID: 19837031 PMC2852643

[6] Chen G Zhang YQ Qadri YJ Serhan CN Ji RR. Microglia in pain: detrimental and protective roles in pathogenesis and resolution of pain. Neuron. Dec 19. (2018) 100:1292–311. doi: 10.1016/j.neuron.2018.11.009, PMID: 30571942 PMC6312407

[7] Inoue K Tsuda M. Microglia in neuropathic pain: cellular and molecular mechanisms and therapeutic potential. Nat Rev Neurosci Mar. (2018) 19:138–52. doi: 10.1038/nrn.2018.2, PMID: 29416128

[8] Guan Z Kuhn JA Wang X Colquitt B Solorzano C Vaman S. Injured sensory neuron-derived CSF1 induces microglial proliferation and DAP12-dependent pain. Nat Neurosci Jan. (2016) 19:94–101. doi: 10.1038/nn.4189, PMID: 26642091 PMC4703328

[9] Malcangio M Sideris-Lampretsas G. How microglia contribute to the induction and maintenance of neuropathic pain. Nat Rev Neurosci May. (2025) 26:263–75. doi: 10.1038/s41583-025-00914-5, PMID: 40128335

[10] Sorge RE Mapplebeck JC Rosen S Beggs S Taves S Alexander JK. Different immune cells mediate mechanical pain hypersensitivity in male and female mice. Nat Neurosci Aug. (2015) 18:1081–3. doi: 10.1038/nn.4053, PMID: 26120961 PMC4772157

[11] Tanaka T Okuda H Isonishi A Terada Y Kitabatake M Shinjo T. Dermal macrophages set pain sensitivity by modulating the amount of tissue NGF through an SNX25-Nrf2 pathway. Nat Immunol Mar. (2023) 24:439–51. doi: 10.1038/s41590-022-01418-5, PMID: 36703006 PMC9977679

[12] Yu X Liu H Hamel KA Morvan MG Yu S Leff J. Dorsal root ganglion macrophages contribute to both the initiation and persistence of neuropathic pain. Nat Commun Jan 14. (2020) 11:264. doi: 10.1038/s41467-019-13839-2, PMID: 31937758 PMC6959328

[13] Ji RR Chamessian A Zhang YQ. Pain regulation by non-neuronal cells and inflammation. Science. Nov 4. (2016) 354:572–7. doi: 10.1126/science.aaf8924, PMID: 27811267 PMC5488328

[14] Luo X Chen O Wang Z Bang S Ji J Lee SH. IL-23/IL-17A/TRPV1 axis produces mechanical pain via macrophage-sensory neuron crosstalk in female mice. Neuron. Sep 1. (2021) 109:2691–2706.e5. doi: 10.1016/j.neuron.2021.06.015, PMID: 34473953 PMC8425601

[15] Jain A Gyori BM Hakim S Jain A Sun L Petrova V. Nociceptor-immune interactomes reveal insult-specific immune signatures of pain. Nat Immunol Jul. (2024) 25:1296–305. doi: 10.1038/s41590-024-01857-2, PMID: 38806708 PMC11224023

[16] Borsook D Hargreaves R Bountra C Porreca F. Lost but making progress–Where will new analgesic drugs come from? Sci Transl Med. (2014) 6:249sr3. doi: 10.1126/scitranslmed.3008320, PMID: 25122640 PMC4319979

[17] Yezierski RP Hansson P. Inflammatory and neuropathic pain from bench to bedside: what went wrong? J Pain. Jun. (2018) 19:571–88. doi: 10.1016/j.jpain.2017.12.261, PMID: 29307749

[18] Sadler KE Mogil JS Stucky CL. Innovations and advances in modelling and measuring pain in animals. Nat Rev Neurosci Feb. (2022) 23:70–85. doi: 10.1038/s41583-021-00536-7, PMID: 34837072 PMC9098196

[19] Liu Y Shen T Li Q Yu X Liu Y Zhou C. Various gases for the treatment of neuropathic pain: mechanisms, current status, and future perspectives. Med Gas Res Dec 1. (2025) 15:488–95. doi: 10.4103/mgr.MEDGASRES-D-24-00161, PMID: 40300884 PMC12124698

[20] O’Connor AB Schwid SR Herrmann DN Markman JD Dworkin RH. Pain associated with multiple sclerosis: systematic review and proposed classification. Pain. Jul. (2008) 137:96–111. doi: 10.1016/j.pain.2007.08.024, PMID: 17928147

[21] Kahraman T Ozdogar AT Ertekin O Ozakbas S. Frequency, type, distribution of pain and related factors in persons with multiple sclerosis. Mult Scler Relat Disord Feb. (2019) 28:221–5. doi: 10.1016/j.msard.2019.01.002, PMID: 30623861

